# The Impact of Antidiabetic Therapy on Liver Injury, Steatosis, and Fibrosis in Patients with Type 2 Diabetes and Metabolic Dysfunction-Associated Steatotic Liver Disease

**DOI:** 10.3390/medicina61101850

**Published:** 2025-10-15

**Authors:** Oana Albai, Adina Braha, Romulus Timar, Sandra Lazăr, Simona Popescu, Bogdan Timar

**Affiliations:** 1 Department of Second Internal Medicine—Diabetes, Nutrition, Metabolic Diseases, and Systemic Rheumatology, “Victor Babes” University of Medicine and Pharmacy, 300041 Timisoara, Romania; albai.oana@umft.ro (O.A.); timar.romulus@umft.ro (R.T.); popescu.simona@umft.ro (S.P.); bogdan.timar@umft.ro (B.T.); 2 Department of Diabetes, Nutrition and Metabolic Diseases Clinic, “Pius Brînzeu” Emergency Clinical County University Hospital, 300723 Timisoara, Romania; 3 Centre for Molecular Research in Nephrology and Vascular Disease, MOL-NEPHRO-VASC, “Victor Babes” University of Medicine and Pharmacy, 300041 Timisoara, Romania; sandra.lazar@umft.ro; 4 Doctoral School of Medicine, “Victor Babes” University of Medicine and Pharmacy, 300041 Timisoara, Romania; 5 First Department of Internal Medicine, “Victor Babes” University of Medicine and Pharmacy, 300041 Timisoara, Romania; 6 Department of Hematology, Emergency Municipal Hospital, 300254 Timisoara, Romania

**Keywords:** MASLD, hepatic steatosis, fibrosis, metabolic syndrome, insulin resistance, type 2 diabetes

## Abstract

*Background and Objectives*: Metabolic dysfunction-associated steatotic liver disease (MASLD) is closely linked with type 2 diabetes mellitus (T2D) and obesity. Despite its growing prevalence, effective pharmacological interventions remain limited, with antidiabetic agents such as glucagon-like peptide-1 receptor agonists (GLP-1 RA) and sodium-glucose cotransporter-2 inhibitors (SGLT2i) showing emerging promise. This study aimed to evaluate the impact of different antidiabetic therapies on hepatic steatosis, fibrosis, and cardiometabolic risk factors in patients with T2D and MASLD from Romania. *Materials and Methods*: We conducted a prospective observational study involving 256 patients with T2D and MASLD followed up for 6 months. Assessed parameters included anthropometry, glycemic indices, lipid profile, renal function, liver enzymes, and non-invasive evaluation of hepatic steatosis and fibrosis. Patients were 53% women, had a median age of 63 years, a median BMI of 32.2 kg/m^2^, a median baseline CAP of 281 dB/m, a FibroScan of 8.9 kPa, and an HbA1c of 8.0%. *Results*: CAP decreased significantly from 281 to 245 dB/m, *p* < 0.0001; FibroScan from 8.9 to 8.0 kPa, *p* < 0.0001. The largest changes were observed in the GLP-1 RA subgroup (CAP −50 dB/m, FibroScan −1.0 kPa, weight −8.0 kg, HbA1c −0.7%), and in the SGLT2i subgroup (CAP −30.5 dB/m, FibroScan −0.7 kPa, weight −4.0 kg, HbA1c −0.5%). In regression analysis, independent factors associated with CAP improvement included GLP-1 RA therapy (β = 44.5, 95% CI 38.3–50.6, *p* < 0.0001), SGLT2i therapy (β = 23.4, 95% CI 15.7–31.1, *p* < 0.0001), and ≥10% weight loss (β = 23.2, 95% CI 12–34.4, *p* < 0.0001). For FibroScan improvement, GLP-1 RA (β = 1.0, 95% CI 0.8–1.2, *p* < 0.0001) and SGLT2i (β = 0.5, 95% CI 0.3–0.7, *p* < 0.0001) therapies were both significant. *Conclusions*: Antidiabetic therapy, particularly GLP-1 RA, was significantly associated with improvement in hepatic steatosis, fibrosis, and cardiometabolic risk in T2D patients with MASLD beyond the weight reduction effect. However, weight loss and lipid modulation enhance these benefits, supporting the development of integrated therapeutic strategies for this high-risk population.

## 1. Introduction

Metabolic Dysfunction-Associated Steatotic Liver Disease (MASLD), formerly termed nonalcoholic fatty liver disease (NAFLD), is an increasingly prevalent condition worldwide, associated with significant morbidity and mortality from metabolic, cardiovascular, and neoplastic diseases. MASLD remains often underdiagnosed, and its etiopathogenesis, although extensively studied, is still not fully elucidated. Because the disease can progress to end-stage liver disease and is frequently associated with extrahepatic manifestations, it is now recognized as a multisystem disorder that requires a multidisciplinary and multifactorial approach [1].

Epidemiological data suggest that MASLD affects nearly one-third of the global population, with prevalence rates of 25–35% in the general population and 50–60% among high-risk groups such as patients with diabetes, obesity, or dyslipidemia. The global prevalence of metabolic dysfunction–associated steatohepatitis (MASH), its more severe inflammatory form, is estimated at approximately 5% [2,3,4].

The burden of MASLD extends beyond liver-related morbidity. Pathophysiologically, MASLD is characterized by hepatic steatosis, confirmed either by imaging or histology, in the presence of metabolic risk factors. Among these, type 2 diabetes mellitus (T2D) and obesity are the most prominent [5]. MASLD is considered the hepatic manifestation of metabolic syndrome.

The relationship between MASLD and T2D is complex and bidirectional. Both conditions share overlapping molecular pathways and genetic predispositions. Crosstalk between hepatocytes, adipocytes, and inflammatory cells further explains how insulin resistance contributes to systemic inflammation, dysregulated lipid/glucose metabolism, and cell injury [6,7]. Elevated expression of growth differentiation factor-15 in adipose tissue and hepatocytes is particularly pronounced in T2D and obesity [7]. Moreover, extracellular vesicles derived from steatotic hepatocytes can influence pancreatic β-cell mass via insulin-mediated pathways, highlighting a novel mechanism of organ crosstalk [8].

Clinically, MASLD progresses from simple steatosis to steatohepatitis, hepatocellular ballooning, fibrosis, and ultimately cirrhosis, liver failure, or hepatocellular carcinoma. Fibrosis, characterized by collagen-rich scar tissue replacing normal parenchyma, is the main predictor of adverse outcomes [9,10,11]. Even though MASLD pathophysiology has been extensively studied, difficulties persist in terms of early diagnosis and optimal management, due to the lack of validated pharmacological therapies and the underdiagnosis of the disease.

In the literature, studies have highlighted the distinct benefits of glucagon-like peptide-1 receptor agonists (GLP-1 RAs) and sodium-glucose cotransporter-2 inhibitors (SGLT2is) on hepatic and cardiometabolic markers. Although both GLP-1 RAs and SGLT2i induce weight loss, these classes exert distinct effects on the liver: GLP-1 RAs exhibit direct anti-inflammatory and antifibrotic actions, while SGLT2i influence hepatic homeostasis and metabolites through renal and hemodynamic mechanisms. However, direct, long-term comparative analyses in real cohorts of patients with MASLD and T2D are lacking. Moreover, most data come from randomized trials with strict selection criteria, with few observational studies validating these effects in real-life settings, such as hospitalized patients with suboptimal glycemic control [12,13]. However, there are no prospective, long-term comparative analyses that highlight the real differences in efficacy in preventing liver steatosis and fibrosis complications between these pharmacological classes. Monotherapy is often insufficient, and combination regimens targeting multiple pathogenic pathways—antioxidant, anti-inflammatory, antifibrotic, and hypolipidemic agents—are under investigation [14,15,16,17]. In advanced cases, bariatric surgery or liver transplantation may be necessary [18,19].

Our study addresses this gap by providing a comparative analysis in a head-to-head, real-world, prospective observational cohort study, evaluating independent associations of various antidiabetic therapies with the dynamic changes in liver steatosis and fibrosis in Romanian patients with T2D and MASLD, with a focus on the individual cardiometabolic profile.

## 2. Materials and Methods

### 2.1. Patients

We conducted a prospective, observational study that included 256 patients with T2D, hospitalized in the Diabetes, Nutrition and Metabolic Diseases Clinic of the County Emergency Hospital “Pius Brînzeu” from Timisoara (Romania) between December 2024 and April 2025. Patients were admitted to the hospital with poor glycemic control for treatment reevaluation according to the local standard of care guidelines by their physician. All patients were taking metformin, a statin, and antihypertensive medications at baseline in stable doses. The patients were then evaluated after 6 months to compare changes in the studied outcomes (liver steatosis and fibrosis, cardiometabolic risk factors). The research was conducted in accordance with the Declaration of Helsinki (2013 version). All patients signed the informed consent. The Ethics Committee of Timișoara County Emergency Hospital approved the study protocol (512) on 26 November 2024.

Inclusion criteria were: age > 18 years, body mass index (BMI) > 18.4 kg/m^2^, previous diagnosis of T2D, with at least 1 year of diabetes duration, blood pressure (BP) ≥ 130/85 mmHg or specific drug treatment, triglycerides ≥ 150 mg/dL, HDL cholesterol (HDLc) < 40 mg/dL, for men and <50 mg/dL, for women or specific drug treatment for dyslipidemia, and signed informed consent. The rationale for cohorting based on admission with suboptimal glycemic control was to create a homogeneous population with active MASLD in which therapeutic intervention could have maximum short-term, monitorable impact.

Exclusion criteria were: significant alcohol consumption, viral hepatitis, autoimmune hepatitis, hepatic cancer, drug-induced liver disease, or neurological or psychiatric conditions, lost to follow-up, or incomplete study data.

The final cohort consisted of 136 women (53.12%) and 120 men (46.88%), with a median age of 63 years and a median duration of diabetes of 11 years.

### 2.2. Clinical Parameters, Biomarkers, and Non-Invasive Imaging Tests

Patients were questioned, and data were collected regarding the antidiabetic medication, consumption of alcohol or other illegal substances, and smoking.

Beyond lifestyle optimization measures, the therapeutic regimen for the study cohort included oral antidiabetic drugs (Metformin, Sulfonylureas), insulin, GLP-1 RA, and SGLT2i.

Regarding weight status, we collected data on body weight, BMI, and abdominal circumference (waist circumference, expressed in cm) at initial admission and at the 6-month follow-up.

Glycemic control was assessed based on glycemic values (fasting (FG) and 2 h postprandial (PPG)) and glycated hemoglobin A1c (HbA1c). Screening for chronic complications of diabetes was performed routinely: microangiopathy (fundoscopy for the diagnosis and staging of diabetic retinopathy (DR), urinary albumin/creatinine ratio (UACr), estimated glomerular filtration rate (eGFR) for classifying patients into stages of diabetic kidney disease according to the Kidney Disease: Improving Global Outcomes—KDIGO classification); macroangiopathy (electrocardiogram, cardiac ultrasound, cerebral computed tomography, Doppler ultrasound of carotid and peripheral arteries), neuropathies (sensitivity tests, nerve conduction velocity).

Cardiometabolic risk factors were also evaluated, including lipid profile (total cholesterol (TC), serum triglycerides (TG), low-density lipoprotein cholesterol (LDLc), high-density lipoprotein cholesterol (HDLc), presence of hypertension (we analyzed only the systolic blood pressure (SBP)), and other inflammatory markers (C-reactive protein (CRP), fibrinogen, erythrocyte sedimentation rate (ESR)).

Liver function was assessed according to the four syndromes: hepatocytolytic, hepatopriv, inflammatory, and bilioexcretory.

Hepatic steatosis was evaluated using the Fibroscan Echosens (France) device, which employs transient elastography and controlled attenuation parameter (CAP), a non-invasive method expressed in decibels per meter (dB/m). A CAP score < 238 dB/m indicates normal fat content (<5%). Steatosis is graded as follows: S1 (mild): 238–260 dB/m, involving 11–33% of hepatocytes; S2 (moderate): 260–290 dB/m, involving 34–66% of hepatocytes; S3 (severe): 290–400 dB/m, involving >67% of hepatocytes [20]. Liver stiffness measurement was expressed in kilopascals (kPa). Normal values range from 2 to 7 kPa. Values ≥ 12.5 kPa are consistent with cirrhosis, characterized by hepatocellular necrosis, connective tissue proliferation, architectural distortion, loss of lobular structure, and the development of regenerative nodules [21,22]. The liver assessments were performed by two independent operators with certified experience, using standard measurement quality criteria (minimum 10 validations, IQR/mean ratio < 30%, exclusion of invalid readings, and no significant variability between the results of the two operators). In cases where values were considered unreliable, measurements were repeated or excluded to ensure the accuracy of liver outcome data.

### 2.3. Statistical Analysis

We conducted a comprehensive statistical analysis using MedCalc^®^ Statistical Software version 23.3.7 (MedCalc Software Ltd., Ostend, Belgium; https://www.medcalc.org; 2025). We summarized the baseline characteristics of the cohort and compared them by gender, using means, medians, standard deviations, 25–75 percentiles according to the variables’ Gaussian distribution, and 95% confidence intervals to provide a detailed view of the cohort’s demographics, anthropometrics, metabolic, hepatic, and cardiovascular status. The distribution of the variables was tested for normality with the Shapiro–Wilk test. To evaluate within-patient longitudinal changes in quantitative outcomes such as liver enzymes (aspartate transaminase (ASAT), alanine transaminase (ALAT)), steatosis (CAP), fibrosis (Fibroscan), glycemic control (HbA1c, FG, PPG), and cardiometabolic risk factors (lipid profile, anthropometry, blood pressure).

In order to evaluate the strength and direction of monotonic relationships between possible associated factors (change in continuous variables from baseline to follow-up at 6 months for each studied parameter) with liver outcomes ∆CAP and ∆Fibroscan we performed a Spearman correlation analysis. Data is presented as a correlogram where the warm colors indicate a positive association (rho (ρ) coefficient closer to +1), while the cold colors indicate a negative association (ρ coefficient closer to −1).

Kruskal–Wallis test (nonparametric ANOVA) with post hoc Conover analysis was employed to compare the degree of change in outcome parameters.

First, we constructed a general multiple linear regression analysis to identify independent predictors of ∆CAP and ∆Fibroscan. Then we constructed multiple linear regression models with the dependent variables ∆CAP and ∆Fibroscan, and independent variables including drug class, weight change (a binary value of ≥10% reduction from baseline), and baseline values of CAP and Fibroscan, to test the strength of association and separate weight-driven effects from direct drug effects. To assess if the effect of the drug differs according to weight loss, we introduced a binary interaction term (drug × 10% weight reduction). The interaction term’s coefficient in regression analysis tests whether the drug effect is modified by weight loss status. If the interaction is statistically significant, then the effect of the drug depends on the weight reduction.

We considered a *p*-value of less than 0.05 to be statistically significant.

## 3. Results

### 3.1. Descriptive Analysis of the Studied Cohort

The present research included 256 patients with T2D and MASLD, with a median age slightly higher in women (65.0 years) compared to men (61.0), a similar median diabetes duration of 11 years, and overweight or obesity, having a median BMI of 32.2 kg/m^2^, with men having a higher mean weight of 95.1 kg compared to women 88.0 kg. The abdominal waist was above the standard threshold according to gender in both men and women, with an overall median of 96 cm. The median baseline HbA1c was 8.0%, with no differences observed by gender. The baseline general characteristics of the studied patients, compared by gender, are presented in Table S1 from the Supplementary Materials.

There was no significant difference in CAP or FibroScan results by gender. Median values of CAP place the cohort firmly in the S2 to S3 categories (median 281 dB/m). Most patients had mild to moderate fibrosis, with a median liver stiffness measurement of 8.9 kPa (25–75 percentiles: 7.4–10.4 kPa).

### 3.2. Analysis of the Dynamics of the Studied Factors

To compare the dynamics of key metabolic, hepatic, and anthropometric parameters in our cohort, we applied the Wilcoxon test (paired samples). The results are presented in Table S2 from the Supplementary Materials. After 6 months of standard of care therapy, patients presented a significant reduction in liver transaminases (ΔALAT = −11.0; ΔASAT = −9.0, *p*  <  0.0001), liver steatosis (ΔCAP = −36.0; *p*  <  0.0001), liver fibrosis (ΔFibroscan = −0.8; *p*  <  0.0001), a significant improvement of lipid profile (ΔTC = −34.5, ΔHDLc = +3.0, ΔLDLc = −21.0, ΔTG = −35.0, *p*  <  0.0001 for all), better glycemic control (ΔFG = −20.0, ΔPPG = −19.0, ΔHbA1c = −0.5; *p*  <  0.0001), body composition improvement (ΔWeight = −5.0, Δwaist = −4.0, ΔBMI = −1.8; *p*  <  0.0001), improved renal function (ΔeGFR = +7.0, ΔUACr = −4.0; *p*  <  0.0001) and improved blood pressure (ΔSBP = −5.0; *p*  <  0.0001).

### 3.3. Correlation Analysis Between Changes in the Studied Factors in the Cohort

**Figure 1:**
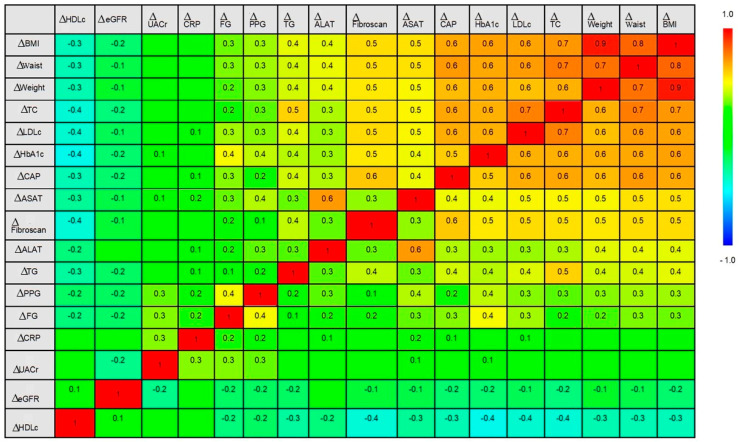
Correlation analysis between the changes in the studied parameters over time.

Figure 1 presents the associations between changes in the studied factors from baseline to follow-up at 6 months in the whole cohort. ∆CAP reduction was highly correlated with: ΔBMI (ρ = 0.6, *p* < 0.0001), ΔWaist circumference (ρ = 0.6, *p* < 0.0001), ΔWeight (ρ = 0.6, *p* < 0.0001), ΔTC (ρ = 0.6, *p* < 0.0001), ΔLDLc (ρ = 0.6, *p* < 0.0001), ΔHbA1c (ρ = 0.5, *p* < 0.0001), and ΔFibroscan (ρ = 0.6, *p* < 0.0001). As CAP decreases, HDLc increases, meaning better cardiometabolic status (ΔHDLc (ρ = −0.3, *p* < 0.0001)). ∆Fibroscan reduction is significantly correlated with: ΔBMI (ρ = 0.5, *p* < 0.0001), ΔWaist (ρ = 0.5, *p* < 0.0001), ΔWeight (ρ = 0.5, *p* < 0.0001), ΔTC (ρ = 0.5, *p* < 0.0001), ΔLDLc (ρ = 0.5, *p* < 0.0001), ΔHbA1c (ρ = 0.5, *p* < 0.0001), and ΔCAP (ρ = 0.6, *p* < 0.0001). As liver stiffness improves, HDLc increases (ρ = −0.4, *p* < 0.0001).

### 3.4. Comparison of the Dynamics of the Studied Parameters According to the Antidiabetic Medication

Table 1 presents a comparative analysis of changes in metabolic, hepatic, and anthropometric parameters over six months, stratified by antidiabetic medication class (GLP-1 RA, SGLT2i, insulin, Other Therapies). The results showed that patients in GLP-1 RA subgroup presented the largest absolute reductions across most parameters, including transaminases (ALAT, ASAT), CAP (−50 dB/m), Fibroscan (−1.0 kPa), lipids, weight (−8 kg), waist (−6 cm), BMI (−2.8 kg/m^2^), and glycemic control (HbA1c −0.7%), compared to the other subgroups treated with SGLT2i, insulin or other therapies. In the SGLT2i subgroup, we also observed significant but generally smaller improvements (CAP −30.5 dB/m; Fibroscan −0.7 kPa; weight −4 kg) than in the GLP-1 RA subgroup. The smallest changes in hepatic and metabolic parameters were observed in the insulin subgroup. In the subgroup treated with other therapies, the improvements in liver and metabolic markers were modest or minimal, and in some cases, even worsened.

**Figure 2:**
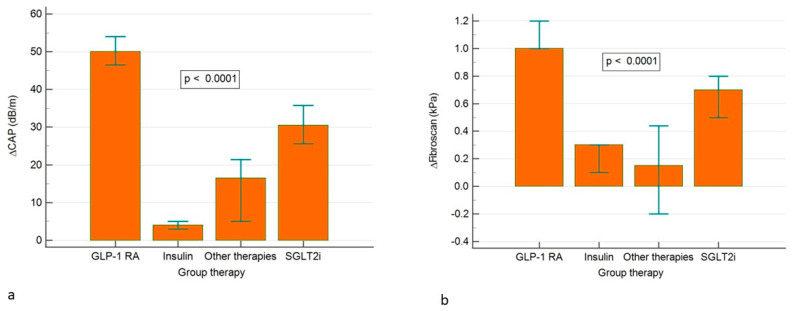
Multiple comparison graph with differences between therapy subgroups of ∆CAP (**a**) and ∆Fibroscan (**b**).

Figure 2 suggests that GLP-1 RA therapy yields the most pronounced improvements in hepatic steatosis (Figure 2a) and fibrosis (Figure 2b), statistically significant compared to other drug classes.

### 3.5. Regression Analysis for Evaluating Potential Associated Factors with the Change in Liver Steatosis (CAP) and Liver Fibrosis (FibroScan)

To assess how various clinical and biochemical changes from baseline values predict the change in CAP in MASLD patients, a stepwise multiple regression analysis was performed. The modeling approach retained only variables that remained statistically significant (*p*  <  0.05): diabetes duration, ∆HDLc, and baseline weight (negative association), ∆waist, ∆CRP, ∆LDLc, ∆SBP, baseline CAP, and baseline waist (positive association). Table 2 presents the coefficients and the effect size of each potential predictive factor. The regression model identified factors significantly associated with the variation in the ΔCAP score, explaining 58.6% of this variation in the analyzed cohort, with a multiple correlation coefficient of 0.7 (F-ratio = 38.7, *p* < 0.0001).

The clinical significance of the results presented in Table 2 is that a longer diabetes duration reduces ΔCAP improvement, leads to a greater decrease in waist circumference, and is associated with a reduction in CRP (indicating systemic inflammation), as well as a decrease in SBP, which predicts more CAP improvement. An increase in HDL is weakly associated with less improvement in CAP (t = −2.0, *p* = 0.03). A decrease in LDLc from baseline is positively associated with greater ΔCAP. A higher baseline CAP and a higher baseline waist circumference predict a greater improvement in CAP. However, the higher the baseline waist, the lower the improvement in CAP.

To assess how various clinical and biochemical changes from baseline values predict changes in Fibroscan in MASLD patients, we performed a stepwise multiple regression analysis. The modeling approach retained only variables that remained statistically significant (*p*  <  0.05): ∆Weight, ∆BMI, ∆HDLc, and ∆LDLc. Table 3 presents the coefficients and the effect size of each potential predictive factor. The model explains 33% of the variance in ∆Fibroscan—a strong explanatory value for clinical research, with a multiple correlation coefficient of 0.5 (F-ratio = 32.3, *p* < 0.0001).

The clinical significance of the results presented in Table 3 is that each kilogram lost predicts a 0.087 kPa improvement in Fibroscan, indicating that greater weight loss is associated with more significant fibrosis regression. The association with ∆BMI also reflects an impact on body composition. An increase in HDL is associated with greater regression of fibrosis. A greater reduction in LDLc may also predict a more favorable change in liver fibrosis, although the effect is small. The 10% weight loss factor was not included in the analysis, likely because continuous changes in weight appear more sensitive than categorical thresholds in this population.

Table 4 presents the results of a multiple regression analysis examining changes in CAP and Fibroscan, as well as significant weight loss (≥10%), in response to different antidiabetic medications.

In model 1 of multiple regression analysis, the coefficient for the therapy with GLP-1 RA is 36.2 (95% CI: 30.3–42.0, *p* < 0.0001), meaning patients treated with GLP-1 RA had an average improvement in CAP of 36.2 dB/ms higher than those not in this group (or compared to the reference), controlling for other factors. This effect is statistically robust (t = 12.1). The R^2^ value is 0.36 (adjusted R^2^ = 0.36), indicating that only about 36% of the variance in CAP improvement is explained by this predictor. The residual standard deviation (23.69) reflects unexplained variability. The overall regression model is statistically significant (F = 147.9, *p* < 0.0001), supporting that the GLP-1 RA effect is a meaningful contributor to CAP change, with a high predictive power. The independent factors, such as 10% weight loss or GLP-1 RA interaction with weight loss, were not retained in the model. From a clinical perspective, these results suggest that GLP-1 RA therapy has a beneficial effect on liver steatosis, extending beyond the weight reduction effect. However, 64% of the changes in CAP are influenced by other factors, not included in this model.

When analyzed with both GLP-1 RA and SGLT2i, in regression model 2, the changes in CAP were improved in 44% of the cases, with 44.5 dB/m higher in the GLP-1 RA group and with 23.4 dB/m higher in the SGLT2i group, beyond the impact of 10% weight loss or drug-weight interaction. When analyzed with SGLT2i alone (model 3), the effect of 10% weight loss, SGLT2i therapy alone, or SGLT2i interaction with weight loss, were not retained in the regression analysis; the improvement in CAP was generally associated with 10% weight loss, through other mechanisms than the ones related to the drug class SGLT2i. However, only 6% of the variance in CAP improvement is explained by 10% weight reduction alone, with a residual standard deviation of 28.8 (*p* = 0.0001).

When analyzed with other therapies than GLP-1 RA or SGLT2i, in regression model 4, the changes in CAP were explained in 18% of the cases by the positive impact of 10% weight loss (coefficient = 19.5, t = 3.6), but more negatively impacted by other therapies (coefficient = −26.3, t = −6.1), with a residual standard deviation of 26.9 (*p* < 0.0001). This means that patients experience less positive change in CAP than patients who lost ≥10% weight in other therapy groups. When other therapies were analyzed together with GLP-1 RA (model 5), patients treated with GLP-1 RA had a 32.7 unit greater reduction in CAP compared to those not on these agents, a highly significant (t = 9.9, *p* < 0.0001), with a positive and robust effect size (partial correlation 0.5).

Regarding the changes in Fibroscan, the results indicated a statistically significant predictive effect of GLP-1 RA, in a model explaining a proportion of 27% of Fibroscan variance in the cohort, with 10% weight loss or GLP-1 RA interaction with weight loss excluded from the model (model 6, F-ratio = 94.8, *p* < 0.0001, residual standard deviation = 0.7).

Model 7 in regression analysis included SGLT2i therapy, 10% weight loss, and the interaction between SGLT2i therapy and 10% weight loss for prediction testing. The analysis ruled out SGLT2i or SGLT2i interaction with weight loss and attributed only the 10% weight loss to 6% of the variance in Fibroscan. Patients who lost 10% of their weight, independent of the effect of SGLT2i, showed an improvement in Fibroscan with 0.6 kPa (F-ratio = 14.2; *p* = 0.0002; residual standard deviation = 0.8).

When analyzed with GLP-1 RA or SGLT2i, in regression model 8, the changes in Fibroscan were explained in 32% of the cases by the positive impact of GLP-1 RA (coefficient 1, t = 11) and SGLT2i (coefficient 0.5, t = 4.4), with a residual standard deviation of 0.7 (*p* < 0.0001). This means that patients treated with GLP-1 RA experience a more positive change in Fibroscan than patients treated with SGLT2i, regardless of the interaction between drug and weight loss.

When other therapies were analyzed together, patients who lost 10% of their weight showed a mean 0.5 kPa improvement in Fibroscan (t = 3.3, *p* = 0.001, model 9). However, the effect was antagonized by the impact of other therapies, which showed an alteration with a mean of 0.7 kPa in Fibroscan (t = −6.0, *p* < 0.0001), with no influence from the drug-weight effect. This model 10 explains 29% of the cases, with a small residual standard deviation.

Our regression analyses showed that both clinical and pharmacological factors were significantly associated with changes in hepatic stiffness and steatosis in patients with MASLD. In the primary model, ∆Weight, ∆BMI, ∆HDLc, and ∆LDLc explained 33% of the variability in Fibroscan measurements (R^2^ = 0.33, F = 32.3, *p* < 0.0001), with a multiple correlation coefficient of 0.5. Each kilogram lost was predicted to result in a 0.087 kPa improvement, underscoring the critical role of continuous weight reduction over categorical thresholds, such as a 10% loss. Improvements in HDLc and reductions in LDLc were independently associated with fibrosis regression.

When other therapies were analyzed together with GLP-1 RA (model 10), patients treated with GLP-1 RA had a 0.7 unit greater reduction in Fibroscan compared to those not on these agents, a highly significant (t = 7.5, *p* < 0.0001) result, with a positive and robust effect size (partial correlation 0.4). In this model, the interaction between other therapies and weight loss, as well as the interaction between GLP-1 and weight loss or 10% weight loss, was not retained in the model (coefficient of determination R^2^ = 0.29, F-ratio = 27.5, *p* < 0.0001, residual standard deviation = 0.7). This suggests that, in the context of the model and the data, weight loss alone did not have an independent effect, but the combination with GLP-1RA did.

## 4. Discussion

At the baseline of the present study, patients had advanced steatosis (median CAP 281 dB/m, S2–S3) and mild to moderate fibrosis (median FibroScan 8.9 kPa), with poor glycemic control (HbA1c 8.0%) and obesity (median BMI 32.2 kg/m^2^). Follow-up analysis showed a marked improvement in liver injury markers: ALAT decreased from 54.5 to 40.0 U/L and ASAT from 52.0 to 38.5 U/L (both *p* < 0.0001). CAP reduced by −36 dB/m (281 → 245 dB/m, *p* < 0.0001) and FibroScan by −0.8 kPa (8.9 → 8.0, *p* < 0.0001), indicating improved steatosis and fibrosis. Metabolic outcomes were equally significant: HbA1c declined from 8.0% to 7.4% (Δ −0.5, *p* < 0.0001), fasting glucose from 162.0 to 148.5 mg/dL, and BMI from 32.2 to 30.1 kg/m^2^ (Δ −1.8, *p* < 0.0001). Lipid profile improved with LDLc reduction from 133.5 to 100.0 mg/dL and triglycerides from 197.5 to 168.5 mg/dL, while HDLc increased from 39.0 to 43.0 mg/dL (all *p* < 0.0001). Correlation analysis showed that reductions in CAP and FibroScan were strongly associated with weight loss, improved HbA1c, and lipid lowering (ρ = 0.5–0.6, *p* < 0.0001), highlighting the interaction between metabolism and the liver in the progression of MASLD.

This observational study revealed significant associations between the use of modern antidiabetic therapies and changes in non-invasive liver markers (CAP, FibroScan) in patients with T2D and MASLD from a Romanian center, who were followed for a short period of 6 months. Our results showed that, within the analyzed cohorts, only certain classes of medication (GLP-1 RA and SGLT2i) were associated with statistically significant improvements in steatosis and cardiometabolic parameters.

Over the 6-month follow-up, patients treated with GLP-1 RA showed the greatest mean improvements in steatosis (CAP −50 dBm) and fibrosis (FibroScan −1.0 kPa), a finding also noted for the SGLT2i subgroup, but with smaller magnitudes. Clinically relevant weight loss (−8.0 kg), waist reduction (−6 cm), and BMI reduction (−2.8 kg/m^2^) were also observed. Glycemic control improved with a mean HbA1c of −0.7%, which was greater than in all other therapy groups. The SGLT2i subgroup also showed significant improvements in CAP (−30.5 dB/m), Fibroscan (−0.7 kPa), LDLc (−22 mg/dL), TG (−28 mg/dL), and body weight (−4.0 kg). These findings are consistent with the recognized metabolic and cardiometabolic benefits of SGLT2i. However, their effect on hepatic markers was less pronounced than that of GLP-1 RA. Additionally, the data presented reflect statistical correlations within the analyzed cohorts, and any ‘effect’ relationship should not be interpreted as evidence of causality in the absence of a randomized experimental design.

Incretin mimetics, beyond their beneficial effect on glycemic control, also demonstrate other metabolic benefits, with increasingly clear results on liver damage, through the remission of steatohepatitis and liver fibrosis. Real-life controlled trials (RCTs) have shown that GLP-1 ARs such as semaglutide, liraglutide, and exenatide used in patients with T2D reduce steatosis measured by magnetic resonance imaging, slow the progression to cirrhosis, and decrease mortality [13,23,24]. The beneficial effects of incretin agonists on hepatic steatosis and metabolic parameters have been demonstrated, with a higher level of evidence, in randomized clinical trials such as LEAN [12] and ESSENCE [25], where liraglutide or semaglutide caused MASH remission and, partially, fibrosis regression, although with more modest histological results for fibrosis compared to steatohepatitis remission. A recent meta-analysis shows that, compared to a placebo, GLP-1 RAs increase the chance of MASH remission by more than four times. However, the absolute reduction in steatosis and improvement in fibrosis on non-invasive imaging are modest, and the long-term clinical significance remains to be proven.

Tirzepatide, a dual agonist (GLP-1/GIP), reduced hepatic steatosis in patients with T2D, demonstrating beneficial effects in the treatment of MASLD. A recent study evaluated the results of Tirzepatide on MASH resolution over 52 weeks of treatment. MASH resolution was achieved in 73.9% of patients receiving the highest dose [26].

Insulin therapy has a positive impact by reducing hepatic steatosis and liver enzyme levels in patients with MASLD [27,28,29,30]. A randomized clinical trial compared insulin glargine with liraglutide and found that both reduced the percentage of hepatocyte fat loading [30].

At the same time, treatment with SGLT2i has led to significant reductions in elastography steatosis in 24-week RCTs; however, the impact on fibrosis and liver complications remains uncertain [27,28]. Glucose uptake by the liver stimulates de novo lipogenesis, so inhibiting glucose reabsorption and increasing its elimination could be extremely beneficial in MASLD. Beyond their effects on glycemic control, SGLT2i reduces hepatic steatosis, IR, liver enzymes, and inflammatory syndrome [31,32,33,34].

Metformin remains a cornerstone therapy for diabetes, but it provides only modest, inconsistent direct benefits on histologic MASH endpoints. However, it is weight-neutral/weight-lowering in some cohorts, inexpensive, safe, and recommended for glycaemic control; its liver-specific utility is limited compared with GLP-1 RA or SGLT2i [29,30,35,36].

The magnitude of the improvements in liver function observed in our study falls within the ranges reported by clinical trials with non-histological follow-up; however, a true regression of fibrosis cannot be concluded, only a short-term association. The clinical experience revealed in this cohort supports the idea that antidiabetic agents that also have a beneficial action on weight (GLP-1 RA, SGLT2i) are associated with a rapid improvement in liver markers, at least in the short term, but randomized trials and long-term comparisons remain essential to demonstrate the real benefit on MASLD progression and liver survival. Current international guidance emphasizes lifestyle as the first-line therapy and suggests the preferential use of weight-lowering (GLP-1 RA, SGLT2i) or weight-neutral (metformin) antihyperglycemics in MASLD, while noting that evidence continues to evolve and that individualized risk–benefit assessment is essential. Ongoing large trials and post-marketing surveillance will determine whether the histologic and clinical liver benefits are associated with deterioration of hepatic function and mortality.

### Study Limitations and Strengths

One of the main strengths of this study is its prospective design, which allows the assessment of the dynamics of hepatic and metabolic parameters after the initiation or modification of antidiabetic treatment. The use of validated methods such as transient elastography (FibroScan) and CAP ensures a rigorous and objective measurement of hepatic steatosis and fibrosis. In addition, the selection of a clinically relevant sample of patients with T2D and MASLD, often with multiple comorbidities and increased cardiovascular risk, increases the applicability of the results to current medical practice.

However, we acknowledge a limitation in the selection of hospitalized patients with uncontrolled diabetes, which reduces the representativeness of the cohorts for the entire MASLD population. This subgroup is clearly distinct in terms of risk and characteristics, limiting the extrapolation of the data. However, we considered that this selection would rather target a subgroup with high cardiovascular/metabolic risk and in need of intensified intervention, where results could have been observed even after a short follow-up period. The 6-month follow-up period may be insufficient to capture the long-term effects of antidiabetic therapies and weight loss on the progression of hepatic fibrosis and steatosis. Longer-term studies are needed to determine whether the observed benefits are sustained over time. The study design was observational and unicentric, which does not allow the establishment of causal relationships, but only the observation of significant associations. However, the purpose of observational cohorts remains to generate robust hypotheses and describe associations relevant to practice, not to demonstrate causality. Lifestyle changes (diet, physical activity) applied by patients during the study follow-up period were not controlled or quantified, so their influence on the observed hepatic and metabolic outcomes cannot be excluded. The absence of a randomized control group limits the ability to attribute the effects of the treatment under study exclusively. The effects may be influenced by concomitant treatments, which are uncontrolled within the observational design. Subgroup analysis, while providing useful insights, is susceptible to selection and prescribing bias due to the uneven distribution of patients and potential differences in baseline profiles between treatment groups. Therefore, we cannot support a causal superiority relationship between therapeutic classes, as the observational design and lack of propensity-score matching imply a risk of residual confounding. The reduction in liver fibrosis by an average of 0.8 kPa, although statistically significant, should be interpreted with caution and cannot be considered to have certified clinical relevance without histological validation.

## 5. Conclusions

This study identified significant associations between the use of certain classes of antidiabetic drugs and improvements in liver parameters and metabolic control in patients with T2D and MASLD. The analysis suggests that the use of GLP-1 RAs and SGLT2i was associated with the most consistent benefits on these parameters; however, these results should be interpreted with caution, given the observational design and potential uncontrolled confounders. The observed effects cannot be considered as evidence of a causal relationship. Further, randomized trials are needed to confirm the central role of these therapies in the management of MASLD in diabetic patients. Several factors, including baseline liver function, glycemic control, body weight, and adherence to therapy, influence the effectiveness of these drugs. Although lifestyle measures play an important role, carefully selected pharmacological therapy, individualized for each patient, is essential for slowing disease progression and improving quality of life. The results of this study support the idea that prioritizing GLP-1RA therapy could be beneficial in T2DM patients with MASLD.

## Figures and Tables

**Table 1:** Comparison of the dynamics of the studied parameters according to the antidiabetic medication.

Variables	Changes in Values from Baseline to Follow-Up				*p* Values
	GLP-1 RA	SGLT2i	Insulin	Other Therapies	
ALAT (u/L)	15.0 (163.0)	9.0 (102.6)	5.0 (93.3)	13.0 (103.8)	<0.0001
ASAT (u/L)	12.0 (170.6)	7.5 (118.8)	6.0 (98.8)	3.5 (65.6)	<0.0001
CAP (dB/m)	50.0 (184.0)	30.5 (122.0)	4.0 (48.4)	16.5 (72.7)	<0.0001
FIBROSCAN (kPa)	1.0 (174.8)	0.7 (128.2)	0.3 (62.2)	0.7 (76.6)	<0.0001
TC (mg/dL)	56.0 (184.5)	30.5 (120.9)	7.0 (62.1)	6.0 (61.6)	<0.0001
HDLc (mg/dL)	−4.0 (98.7)	−3 (132.6)	−1 (173.2)	2 (156.0)	<0.0001
LDLc (mg/dL)	45.0 (180.2)	22.0 (124.8)	6.0 (72.8)	−10.5 (58.7)	<0.0001
TG (mg/dL)	37.0 (161.6)	28.0 (120.2)	7.0 (62.8)	6.0 (114.3)	<0.0001
PPG (mg/dL)	25.0 (145.1)	31.5 (191.1)	6.0 (78.3)	−18.5 (68.7)	<0.0001
FG (mg/dL)	22.0 (141.1)	36.5 (192.1)	6.0 (69.1)	−23 (84.3)	<0.0001
HbA1c (%)	0.7 (179.1)	0.5 (122.4)	0.2 (64.9)	0.1 (70.1)	<0.0001
Weight (kg)	8.0 (184.7)	4.0 (106.2)	2.0 (68.0)	2.0 (71.1)	<0.0001
Waist (cm)	6.0 (195.8)	3.0 (104.6)	2.0 (67.3)	2.0 (47.6)	<0.0001
BMI (kg/m^2^)	2.8 (186.2)	1.4 (112.1)	0.7 (72.2)	0.4 (58.1)	<0.0001
eGFR (ml/min)	−7.0 (123.1)	−13.5 (95.5)	−6.0 (139.6)	−5.0 (164.7)	<0.0001
UACr (mg/g)	3.0 (121.7)	16.0 (178.6)	3.0 (117.7)	−0.5 (102.8)	<0.0001
SBP (mmHg)	10.0 (173.7)	3.0 (93.0)	3.0 (89.4)	0.0 (91.9)	<0.0001

Kruskal–Wallis test, post hoc analysis (Conover); *p* < 0.05, statistically significant. Abbreviations: BMI, body mass index; HbA1c, glycated hemoglobin; FG, fasting glycemia; PPG, postprandial glycemia; ASAT, Aspartate transaminase; ALAT, Alanine transaminase; LDLc, low-density lipoprotein cholesterol; TC, total cholesterol; TG, triglycerides; HDLc, high-density lipoprotein cholesterol; eGFR, estimated glomerular filtration rate; UACr, urinary albumin/creatinine ratio; SBP, systolic blood pressure; CAP, controlled attenuation parameter.

**Table 2:** Multiple linear regression analysis of changes in CAP and potential predictive factors.

Independent Variables	Coefficient	Std. Error	95% CI	t	*p*	r_partial_	r_semipartial_
(Constant)	−72.1	14.5	−100.7–−43.6	−4.9	<0.0001		
Diabetes duration (years)	−0.7	0.1	−1.0–−0.3	−3.7	0.0002	−0.2	0.1
∆waist (cm)	2.2	0.5	1.2–3.2	4.3	<0.0001	0.2	0.1
∆CRP (mg/L)	0.4	0.1	0.1–0.82	2.8	0.005	0.1	0.1
∆HDLc (mg/dL)	−0.3	0.1	−0.6–0.0	−2.0	0.03	−0.1	0.0
∆LDLc (mg/dL)	0.1	0.0	0.0–0.2	3.2	0.001	0.2	0.1
∆SBP (mmHg)	0.2	0.1	0.0–0.4	2.1	0.03	0.1	0.0
Baseline CAP (dB/m)	0.2	0.0	0.1–0.2	7.3	<0.0001	0.4	0.3
Baseline weight (kg)	−0.3	0.1	−0.6–0.0	−2.3	0.01	−0.1	0.0
Baseline waist (cm)	0.7	0.1	0.3–1.0	3.9	0.0001	0.2	0.1

Stepwise multiple linear regression model, coefficient of determination R^2^ = 0.58, residual standard deviation 19.4, F-ratio 38.6, *p* < 0.0001. The following variables were not included in the model: age, 10% weight loss, ∆Weight, ∆BMI, ∆HbA1c, ∆PPG, ∆FG, ∆TC, ∆TG, ∆ASAT, ∆ALAT, ∆eGFR, ∆UACr, and baseline HbA1c. Abbreviations: CRP, C-reactive protein; BMI, body mass index; HbA1c, glycated hemoglobin; FG, fasting glycemia; PPG, postprandial glycemia; ASAT, Aspartate transaminase; ALAT, Alanine transaminase; LDLc, low-density lipoprotein cholesterol; TG, triglycerides; HDLc, high-density lipoprotein cholesterol; eGFR, estimated glomerular filtration rate; UACr, urinary albumin/creatinine ratio; SBP, systolic blood pressure; CAP, controlled attenuation parameter; *p* < 0.05, statistically significant.

**Table 3:** Multiple linear regression analysis of changes in Fibroscan and potential predictive factors.

Independent Variables	Coefficient	Std. Error	95% CI	t	*p*	r_partial_	r_semipartial_
(Constant)	0.2	0.0	0.1–0.3	3.9	0.0001		
∆Weight	0.0	0.0	−0.1–0.0	−2.4	0.01	−0.1	0.1
∆BMI	0.4	0.1	0.2–0.6	4.0	0.0001	0.2	0.2
∆HDLc	0.0	0.0	0.0–0.0	−3.1	0.0020	−0.1	0.1
∆LDLc	0.0	0.0	0.0–0.0	3.7	0.0002	0.2	0.1

Stepwise multiple regression model, coefficient of determination R^2^ = 0.33, residual standard deviation 0.7, F-ratio = 32.3, *p* < 0.0001. The following variables were not included in the model: age, diabetes duration, 10% weight loss, ∆HbA1c, ∆PPG, ∆FG, ∆waist (cm), ∆SBP, ∆CRP, ∆TC, ∆TG, ∆ASAT, ∆ALAT, ∆eGFR, ∆UACr, baseline HbA1c, baseline CAP, baseline weight, and baseline waist. Abbreviations: BMI, body mass index; HbA1c, glycated hemoglobin; FG, fasting glycemia; PPG, postprandial glycemia; ASAT, Aspartate transaminase; ALAT, Alanine transaminase; LDLc, low-density lipoprotein cholesterol; TG, triglycerides; HDLc, high-density lipoprotein cholesterol; eGFR, estimated glomerular filtration rate; UACr, urinary albumin/creatinine ratio; SBP, systolic blood pressure; CAP, controlled attenuation parameter; *p* < 0.05, statistically significant.

**Table 4:** Multiple linear regression analysis of changes in CAP and Fibroscan and significant weight loss with different antidiabetic medications.

Models	Independent Variables	Coefficient	Std. Error	95% CI	t	*p*	r_partial_	r_semipartial_
∆CAP Model 1	10% weight loss or GLP-1 RA interaction with weight loss were not retained in the model; coefficient of determination R^2^ = 0.36, F-ratio = 147.9, *p* < 0.0001; residual standard deviation = 23.69.							
	(Constant)	17.7	1.9	13.8–21.6	8.9	<0.0001		
	GLP-1 RA	36.2	2.9	30.3–42.0	12.1	<0.0001	0.6	0.6
∆CAP Model 2	10% weight loss or GLP-1 RA interaction with weight loss, or SGL2i interaction with weight loss were not retained in the model; coefficient of determination R^2^ = 0.44, F-ratio = 102.0, *p* < 0.0001, residual standard deviation = 22.2.							
	(Constant)	9.4	2.3	4.8–14.0	4.0	0.0001		
	GLP-1 RA	44.5	3.1	38.3–50.6	14.2	<0.0001	0.6	0.6
	SGLT2i	23.4	3.9	15.7–31.1	5.9	<0.0001	0.3	0.2
∆CAP Model 3	SGLT2i therapy alone or SGLT2i interaction with weight loss were not retained in the model; coefficient of determination R^2^ = 0.06, F-ratio = 16.6, *p* = 0.0001, residual standard deviation = 28.8.							
	(Constant)	31.3	1.9	27.6–35.1	16.3	<0.0001		
	10% weight loss	23.2	5.6	12.0–34.4	4.0	0.0001	0.2	0.2
∆CAP Model 4	other therapies factor interaction with weight loss was not retained in the model; coefficient of determination R^2^ = 0.18, F-ratio = 28.5, *p* < 0.0001, residual standard deviation = 26.9.							
	(Constant)	36.9	2.0	33.0–40.9	18.4	<0.0001		
	10% weight loss	19.5	5.3	8.9–30.0	3.6	0.0003	0.2	0.2
	Other therapies	−26.3	4.2	−34.7–−17.9	−6.1	<0.0001	−0.3	0.3
∆CAP Model 5	other therapies interaction with weight loss or GLP-1 RA interaction with weight loss were not retained in the model; coefficient of determination R^2^ = 0.38, F-ratio = 78.1, *p* < 0.0001, residual standard deviation = 23.4.							
	(Constant)	21.2	2.4	16.4–26.1	8.6	<0.0001		
	Other_therapies	−9.8	4.1	−18.0–−1.7	−2.3	0.01	−0.1	0.1
	GLP-1 RA	32.7	3.2	26.2–39.1	9.9	<0.0001	0.5	0.4
∆Fibroscan—Model 6	10% weight loss or GLP-1 RA interaction with weight loss were not retained in the model; coefficient of determination R^2^ = 0.27, F-ratio = 94.8, *p* < 0.0001, residual standard deviation = 0.7.							
	(Constant)	0.3	0.0	0.2–0.5	6.3	<0.0001		
	GLP-1 RA	0.8	0.0	0.7–1.0	9.7	<0.0001	0.5	0.5
∆Fibroscan—Model 7	SGLT2i or SGLT2i interaction with weight loss were not retained in the model; coefficient of determination R^2^ = 0.05, F-ratio = 14.2, *p* = 0.0002, residual standard deviation = 0.8.							
	(Constant)	0.7	0.0	0.6–0.8	13.0	<0.0001		
	10% weight loss	0.6	0.1	0.2–0.9	3.7	0.0002	0.2	0.2
∆Fibroscan—Model 8	10% weight loss or GLP-1 RA interaction with weight loss, or SGL2i interaction with weight loss were not retained in the model; coefficient of determination R^2^ = 0.32, F-ratio = 60.7, *p* < 0.0001, residual standard deviation = 0.7.							
	(Constant)	0.1	0.0	0.0–0.3	2.6	0.009		
	GLP-1 RA	1.0	0.0	0.8–1.2	11.0	<0.0001	0.5	0.5
	SGLT2i	0.5	0.1	0.3–0.7	4.4	<0.0001	0.2	0.2
∆Fibroscan—Model 9	other therapies interaction with weight loss was not retained in the model; coefficient of determination R^2^ = 0.17, F-ratio = 26.4, *p* < 0.0001, residual standard deviation = 0.7.							
	(Constant)	0.8	0.0	0.7–0.9	15.1	<0.0001		
	10% weight loss	0.5	0.1	0.2–0.8	3.3	0.001	0.2	0.1
	Other therapies	−0.7	0.1	−0.9–−0.5	−6.0	<0.0001	−0.3	0.3
∆Fibroscan—Model 10	other therapies interaction with weight loss, GLP-1 interaction with weight loss, or 10% weight loss were not retained in the model; coefficient of determination R^2^ = 0.29, F-ratio = 27.5, *p* < 0.0001, residual standard deviation = 0.7.							
	(Constant)	0.5	0.0	0.3–0.6	6.8	<0.0001		
	Other therapies	−0.3	0.1	−0.6–−0.1	−2.9	0.003	−0.1	0.1
	GLP-1 RA	0.7	0.1	0.5–0.9	7.5	<0.0001	0.4	0.3

## Data Availability

Patients who participated in this study did not provide written consent for the public dissemination of their data; therefore, supporting data are unavailable due to the sensitive nature of the research.

## Supplementary Materials

The following supporting information can be downloaded at: https://www.mdpi.com/article/10.3390/medicina61101850/s1, Table S1: Baseline general characteristics of studied patients, compared by gender; Table S2: Dynamics of studied parameters.
