# Supplementary material for: The Impact of Antidiabetic Therapy on Liver Injury, Steatosis, and Fibrosis in Patients with Type 2 Diabetes and Metabolic Dysfunction-Associated Steatotic Liver Disease
Source: Medicina (Kaunas). 2025 Oct 15;61(10):1850. doi: 10.3390/medicina61101850 (PMC12566187; doi:10.3390/medicina61101850)
Supplement: Supplementary file 1 [file medicina-61-01850-s001.zip › medicina-3913158-supplementary.pdf]

Table S1. Baseline general characteristics of studied patients, compared by gender.

Variables	Women	Men	Overall (25-75 P)	(95% CI)	p value
Age (years)	65.0 ± 10.0	61.0 (57.0 - 69.5)	63.0 (57.0 - 71.0)	61.0 - 65.0	0.1
Diabetes Duration (years)	11.0 (8.0 - 16.0)	10.0 (7.0 - 15.0)	11.0 (7.0 - 15.0)	10.0 - 12.0	0.2
Weight (kg)	88.0 (81.0 - 92.0)	95.1 ± 10.5	90.0 (85.0 - 97.2)	89.0 - 91.7	<0.0001
Waist (cm)	96.0 (90.2 – 100.0)	96.0 (90.0 - 101.0)	96.0 (90.0 - 100.5)	95.0 - 97.0	0.8
BMI (kg/m^2)	32.5 (30.0 - 35.1)	31.9 ± 3.5	32.2 (29.7 - 35.0)	31.7 - 32.7	0.5
ALAT (u/L)	52.0 (40.2 - 67.7)	55.0 (44.7 - 67.0)	54.5 (43.0 - 66.5)	51.0 - 56.0	0.2
ASAT (u/L)	50.0 (35.0 - 60.7)	52.0 (38.0 - 66.0)	52.0 (38.0 - 61.0)	47.0 - 54.0	0.3
CAP (dB/m)	279.0 (258.2 - 297.7)	285.0 (266.5 - 301.2)	281.0 (264.0 - 298.5)	277.0 - 286.1	0.09
FIBROSCAN (kPa)	8.9 ± 2.2	9.0 ± 2.0	8.9 (7.4 - 10.4)	8.3 - 9.2	0.6
ALP	76.0 (61.2 - 94.7)	83.0 (64.7 - 102.0)	78.0 (62.0 - 98.0)	75.8 - 83.0	0.1
GGT (u/L)	56.0 (39.2 - 73.0)	59.0 (37.7 - 81.0)	58.0 (39.0 - 76.5)	52.8 - 61.0	0.4
TB (mg/dl)	1.1 (0.9 - 1.3)	1.1 (0.9 - 1.2)	1.10 (0.9 - 1.3)	1.0 - 1.1	0.6
Cholinesterase (u/L)	4621.0 (3952.0 - 5885.7)	4852.0 (3917.5 - 5867.0)	4768.5 (3952.0 - 5870.0)	4509.3 - 4994.1	0.7
ESR (mm/h)	20.0 (13.0 - 27.0)	21.0 (13.0 - 29.0)	20.0 (13.0 - 28.0)	19.0 - 22.0	0.6
Fibrinogen	327.0 (283.2 - 394.2)	340.0 (284.5 - 428.5)	330.0 (283.0 - 408.5)	306.6 - 352.0	0.2
CPR (mg/l)	9.8 (5.7 - 21.0)	9.0 (7.0 - 18.2)	9.5 (6.0 - 18.5)	9.0 - 11.0	0.6
HbA1c (%)	8.0 (7.5 - 8.5)	8.0 (7.7 - 8.7)	8.0 (7.6 - 8.5)	7.9 - 8.1	0.4
FG (mg/dl)	162.0 (147.0 - 177.7)	163.4 ± 20.2	162.0 (148.5 - 178.0)	160.0 - 166.0	0.8
PPG (mg/dl)	183.2 ± 28.4	186.0 (169.0 - 204.0)	187.0 (167.0 to 204.0)	181.8 - 189.0	0.6
HDLc (mg/dl)	40.2 ± 8.5	38.1 ± 8.7	39.0 (34.0 - 45.0)	38.0 - 40.0	0.1
LDLc (mg/dl)	133.3 ± 54.8	132.0 (98.0 - 185.0)	133.5(98.0 - 179.0)	125.8 - 141.0	0.8
TG (mg/dl)	190.0 (167.7 - 256.0)	205.0 (177.5 - 259.5)	197.5 (173.0 - 258.0)	189.0 - 207.0	0.2
TC (mg/dl)	225.5 ± 55.2	228.0 ± 63.2	228.5 (191.5 - 266.0)	219.8 - 240.0	0.8
Serum Creatinine (mg/dl)	1.0 (0.9 - 1.2)	1.0 (0.8 - 1.4)	1.0 (0.8 - 1.3)	1.0 - 1.1	0.5
eGFR (ml/min)	58.0 (49.2 - 74.0)	78.0 (59.0 - 98.0)	64.0 (53.0 - 87.5)	60.0 - 70.0	<0.0001
UACr (mg/g)	43.3 (30.4 - 67.5)	39.0 (26.9 - 79.7)	42.0 (28.7 - 75.0)	38.8 - 48.0	0.5

SBP (mmHg)	151.3 ± 21.5	155.0 ± 22.3	153.5 (140.0 - 166.5)	150.0 - 155.0	0.5
Smoking status	35.3% (49/139)	47.9% (56/117)	41.0% (105/256)		0.04
DN	49.6% (69/139)	35.9% (42/117)	43.4% (111/256)		0.02
DR	51.1% (71/139)	53.0% (62/117)	52.0% (133/256)		0.7
PAD	43.9% (61/139)	40.2% (47/117)	42.2% (108/256)		0.5
Stroke	16.5% (23/139)	13.7% (16/117)	15.2% (39/256)		0.5
CHD	49.3% (68/139)	48.3% (56/117)	48.8% (125/256)		0.8
GLP-1 RA	43.2% (60/139)	47% (55/117)	44.9% (115/256)		
SGLT2i	21.6% (30/139)	17.1% (20/117)	19.5% (50/256)		
Insulin	15.8% (22/139)	16.2% (19/117)	16.0% (41/256)		0.8
Other therapies	19.4% (27/139)	19.7% (23/117)	19.5% (50/256)		

Mann-Whitney test of T tests for sex differences, depending on the variables' distribution; Continuous variables (with non-Gaussian distribution) are described by their median (25-75 percentiles (P)). Continuous variables (with a Gaussian distribution) are described by their mean and standard deviation. Categorical variables are presented as proportions and absolute numbers (counts); $p < 0.05$, statistically significant.

Abbreviations: P, percentiles; CI, confidence interval; BMI, body mass index; HbA1c, glycated hemoglobin; FG, fasting glycemia; PPG, postprandial glycemia; ASAT, Aspartate transaminase; ALAT, Alanine transaminase; TB, Total bilirubin; GGT, Gamma-glutamyl transferase; ALP, Alkaline phosphatase; ESR, Erythrocyte sedimentation rate; CRP, C-Reactive Protein; LDLc, low-density lipoprotein cholesterol; TG, triglycerides; HDLc, high-density lipoprotein cholesterol; eGFR, estimated glomerular filtration rate; UACr, urinary albumin/creatinine ratio; SBP, systolic blood pressure; CAP, controlled attenuation parameter; DN, diabetic neuropathy; DR, diabetic retinopahty; PAD, peripheral arterial disease; CHD, coronary heart disease; GLP-1 RA, glucagon like-peptide-1 receptor agonists; SGLT2i, sodium-glucose co-transporter-2 inhibitors.

Table S2. Dynamics of studied parameters.

Variables	Baseline	Follow-up	Paired differences	
	Median	Median	Median	p [a]
ALAT (u/L)	54.5	40.0	-11.0	<0.0001
ASAT (u/L)	52.0	38.5	-9.0	<0.0001
CAP (dB/m)	281.0	245.0	-36.0	<0.0001
FIBROSCAN (kPa)	8.9	8.0	-0.8	<0.0001
TC (mg/dl)	228.5	187.0	-34.5	<0.0001
HDLc (mg/dl)	39.0	43.0	3.0	<0.0001
LDLc (mg/dl)	133.5	100.0	-21.0	<0.0001
TG (mg/dl)	197.5	168.5	-35.0	<0.0001
PPG (mg/dl)	187.0	167.0	-19.0	<0.0001
FG (mg/dl)	162.0	148.5	-20.0	<0.0001
HbA1c (%)	8.0	7.4	-0.5	<0.0001
Weight (kg)	90.0	85.0	-5.0	<0.0001
Waist (cm)	96.0	92.0	-4.0	<0.0001
BMI (kg/m^2)	32.2	30.1	-1.8	<0.0001
eGFR (ml/min)	64.0	78.0	7.0	<0.0001

UACr (mg/g)	42.0	38.8	-4.0	<0.0001
SBP (mmHg)	153.5	149.0	-5.0	<0.0001

Wilcoxon test (paired samples).

Abbreviations: BMI, body mass index; HbA1c, glycated hemoglobin; FG, fasting glycemia; PPG, postprandial glycemia; ASAT, Aspartate transaminase; ALAT, Alanine transaminase; LDLc, low-density lipoprotein cholesterol; TG, triglycerides; HDLc, high-density lipoprotein cholesterol; eGFR, estimated glomerular filtration rate; UACr, urinary albumin/creatinine ratio; SBP, systolic blood pressure; CAP, controlled attenuation parameter; $p < 0.05$, statistically significant.

After 6 months of standard of care therapy, patients presented a significant reduction in liver transaminases (ALAT 54.5 → 40.0 u/L, ΔALAT = −11.0; ASAT 52.0 →38.5 u/L, ΔASAT = −9.0, $p < 0.0001$), liver steatosis (CAP 281.0 → 245.0 dB/m, ΔCAP = −36.0; $p < 0.0001$), liver fibrosis (Fibroscan 8.9 → 8.0 kPa, ΔFibroscan = −0.8; $p < 0.0001$), a significant improvement of lipid profile (TC 228.5 → 187.0 mg/dL, ΔTC = −34.5, HDLc 39.0 → 43.0 mg/dL, ΔHDLc = +3.0, LDLc 133.5 → 100.0 mg/dL, ΔLDLc = −21.0, TG 197.5 → 168.5 mg/dL, ΔTG = −35.0, $p < 0.0001$ for all), better glycemic control (FG 162.0 → 148.5 mg/dL, ΔFG = −20.0, PPG 187.0 → 167.0 mg/dL, ΔPPG = −19.0, HbA1c 8.0 → 7.4%, ΔHbA1c = −0.5; $p < 0.0001$), body composition improvement (weight 90.0 → 85.0 kg, ΔWeight = −5.0, abdominal waist 96.0 → 92.0 cm, Δwaist = −4.0, BMI 32.2 → 30.1 kg/m^2, ΔBMI = −1.8; $p < 0.0001$), improved renal function (eGFR 64.0 → 78.0 mL/min, ΔeGFR = +7.0, UACr 42.0 → 38.8 mg/g, ΔUACr = −4.0; $p < 0.0001$) and improved blood pressure (SBP 153.5 → 149.0 mmHg, ΔSBP = −5.0; $p < 0.0001$).
